# Does owning a pet protect older people against loneliness?

**DOI:** 10.1186/1471-2318-14-106

**Published:** 2014-09-20

**Authors:** Jitka Pikhartova, Ann Bowling, Christina Victor

**Affiliations:** Department of Clinical Sciences, College of Health and Life Sciences, Brunel University London, Uxbridge, Middlesex UB8 3PH UK; Faculty of Health Sciences, University of Southampton, Southampton, SO17 1BJ UK

**Keywords:** Loneliness, ELSA, Pet ownership, Longitudinal study, Old people

## Abstract

**Background:**

Pet ownership is thought to make a positive contribution to health, health behaviours and the general well-being of older people. More specifically pet ownership is often proposed as a solution to the problem of loneliness in later life and specific ‘pet based’ interventions have been developed to combat loneliness. However the evidence to support this relationship is slim and it is assumed that pet ownership is a protection against loneliness rather than a response to loneliness. The aim of this paper is to examine the association between pet ownership and loneliness by exploring if pet ownership is a response to, or protection against, loneliness using Waves 0–5 from the English Longitudinal Study of Ageing (ELSA).

**Methods:**

Using data from 5,210 men and women in the English Longitudinal Study of Ageing, cross-sectional and longitudinal regression analysis was used to assess the bi-directional relationship between loneliness and pet ownership among adults aged 50 + .

**Results:**

In 2001 (wave 0) 41% of participants were pet owners compared with 30% in 2010 (Wave 5). The association between pet ownership and loneliness is stronger in women than men, and in both directions (i.e. pet ownership predicting loneliness and loneliness predicting pet ownership) and of the similar magnitude (OR 1.2-1.4). Age, social relationships, demographic factors and health behaviour variables have only a minimal influence upon the association between loneliness and pet ownership. The results of our longitudinal analysis showed that women who reported being lonely always in Waves 0 to 5 were more likely to have a pet in Wave 5.

**Conclusion:**

Reported loneliness is dependent on socio-demographic characteristics such as gender, household income, household living arrangements and health status. Taking those factors into account, owning a pet significantly influences later reporting of loneliness in women in our longitudinal analysis. In the reverse direction, reported loneliness influences pet ownership in later waves. In both directions, the relatively strong gender interaction suggests the association is limited to women with effects for men minimal or non-existent.

## Background

Loneliness in later life, its prevalence and risk factors, has long been a focus of research. According to cognitive discrepancy theory, loneliness is defined as an unwanted discrepancy between desired and achieved levels of social contact [1].

In North America, Australasia and Western Europe research has consistently reported the prevalence of severe loneliness of approximately 10% for those aged 65 years and older with a further 30% classified as moderately lonely whilst countries in Central and Eastern Europe report prevalence rates of severe loneliness of between 15% and 20% [2]. Loneliness has been shown to be associated with a range of negative health outcomes and health behaviours (which vary between different age groups) [3, 4].

Previous research has identified a range of risk factors for the onset of loneliness which also vary somewhat across age groups. Predictors of loneliness in younger ages have been summarized by Mahon et al [5]. A much wider range of risk factors have been identified for older adults including gender [6], being widowed or divorced [7], reporting poorer self-rated health than expected [8, 9], sensory impairments [10], disability/impaired mobility [11], poverty and low material resources [6], time spent alone and household composition [8]. Living arrangements, social resources and social participation have been identified as potential mediators between health status and loneliness [12]. In terms of health outcomes loneliness has been linked with cardiovascular disease [13, 14], depression [15] and Alzheimer disease [16], and has been proposed as a mortality accelerator [17–19]. According to review published by Holt-Lundstad et al. [20] the mortality excess associated with weak social relationships is similar to other established risk factors such as low physical activity, smoking or drinking. Loneliness is, therefore, an important public health issue, and it is thus important to identify factors which can protect against or reduce vulnerability to loneliness as a means of developing appropriate interventions. Some of the established risk factors have been used in interventions to reduce loneliness. Since 2000 at least five reviews and meta-analyses to evaluate the evidence for the effectiveness of interventions to reduce loneliness have been published. Four focussed on older adults (the reviews by Cattan, et al. [21], Findlay [22], Choi et al. [23] and Hagan et al. [24]) whilst Masi et al. [25] included adults of all ages. These reviews showed that only a limited number of interventions demonstrated any significant impact upon levels of loneliness [21].

A number of interventions attempting to prevent or reduce loneliness have used ‘pet therapy’ based upon the attachment theory of Bowlby which emphasized the human need to be attached to somebody, to be close, to form and maintain relationships and the need for a sense of belonging [26]. Such interventions also build on work by Lazarrus and Folkman [27] indicating that human-pet attachment could provide a unique and affordable source of social support [28]. In the UK it is estimated that there are approximately 27 million pets and 45% of British households own a pet (Pet Food Manufacturers Association; http://www.pfma.org.uk/pet-population/; *accessed December 2013*). The UK ranks second in Europe for dog ownership, and third for cat ownership [29]. It is claimed that people who own pets do so to improve their subjective well-being, for company and to feel loved, depended upon and wanted [30].

The evidence base to support the use of such pet-based interventions is weak. There are a number of studies focusing on pet ownership or on animal assisted therapy (AAT) for older adults, for adults with serious mental health problems [31], cardiovascular events [28, 32, 33], or living in care-homes [34] which demonstrate positive outcomes [35–37]. Several studies have explored how, and to what extent, feeling of loneliness and social exclusion can be remediated (or prevented) by pet ownership [38–40] based on the premise that this reduces the impact of stressors in everyday live and consequently symptoms of depression or anxiety [41–44]. It is also hypothesised that pets may substitute for missing attachment figure(s). However, those who are highly attached to their pet report higher levels of loneliness compared to those who do not have such close relationship with their pet [45].

The evidence for the beneficial impact of pet ownership on loneliness is inconsistent as some studies show no impact of pet-ownership on health status or on mortality [46]. Furthermore most studies looking at this relationship are cross sectional in design. There is lack of evidence from longitudinal and intervention studies of using animals to reduce loneliness and social isolation among older people [47] although some effective interventions have been identified [48]. Longitudinal studies examining the relationship between pet-ownership or frequent contact with animals, and health, well-being or loneliness are rare. Raina et al. [49], focusing on the relationship between pet ownership and the physical and mental health of older people, reported that those who owned a pet were more active at the end of the study period than non-pet-owners, and that pet ownership significantly modified the relationship between social support and change in mental health. [49] Guest et al. reported that hearing dogs had a big impact on reducing loneliness among hearing-impaired owners but they did not use any control group [50].

Given that the evidence is mixed and extremely limited, the effectiveness of the presence of home pets on the prevention of loneliness and social isolation and improvement of subjective well-being has been questioned [51–53]. Furthermore rather than pet ownership mediating against loneliness, it has been proposed that the true nature of the relationship is, in fact, reversed i.e. pet ownership is a response to loneliness. However there are few longitudinal studies assessing the relationship between loneliness and pet ownership in both directions (pet ownership as a response to loneliness or pet ownership as a protection against loneliness). [54] The aim of this paper is to contribute to the evidence examining the relationship between pet ownership and loneliness. We examine if pet ownership is a response to, or protection against, loneliness using the English Longitudinal Study of Ageing (ELSA) by considering four questions:

*Question 1*: Can pet ownership protect against future loneliness?

*Question 2*: Is current pet ownership a response to previous feelings of loneliness?

*Question 3*: How do different pathways of loneliness influence current pet ownership?

*Question 4*: What role do socio-demographic characteristics, known to be connected with loneliness, play in the association between pet ownership and loneliness?

The answers to these questions will extend the evidence about the potential long-term effects of the presence of domestic animals in the lives of older people on the feelings of loneliness and vice versa.

## Methods

### Data

The analysis was performed on a subsample of publicly available data from the English Longitudinal Study of Ageing (ELSA). The ELSA dataset is based on the Health Survey for England (HSE) and is designed as a representative sample of the population aged 50+ years of age living in the community in England. Those aged 50+ who participated in the HSE in 1998, 1999 and 2001 (referred to as Wave 0) were invited to participate in the Wave 1 of ELSA in 2002. The study has collected data every two years since 2002 with biological samples taken every 4 years. Participants gave full informed written consent to participate in the study and ethical approval was obtained from the London Multicentre Research Ethics Committee. More details about ELSA can be found at http://www.ifs.org.uk/elsa/documentation.php.

The two possible directions of the association between loneliness and pet ownership (as a protection against or response to loneliness and current pet ownership as a response to previous feelings of loneliness) are explored using two analytical samples. Questions about pet ownership were included in part of Wave 0 and in Wave 5 while questions related to loneliness were first included in Wave 2 and have been presented in all subsequent waves.

To answer Question 1 (does pet ownership protect against loneliness), we consider the relationship between pet-ownership in Wave 0 and loneliness reported in following waves (Waves 2 to 5). For this analysis a subset of 2,141 individuals present in the part of Wave 0 that included pet ownership questions (only 1 of the 3 years of HSE data that formed the original sample for ELSA) and subsequent ELSA waves is used.

To answer question 2 (is pet ownership a response to loneliness), we consider reported pet ownership in Wave 5 (the only other ELSA wave including pet ownership questions) and loneliness reported in Waves 2–5 using both cross-sectional and longitudinal analysis. The cross-sectional analysis is based on data from Wave 5 (as it is the only wave with data both on loneliness and pet ownership). Longitudinal analysis will assess the relationship between loneliness reported in Waves 2 to 5 and pet ownership in the Wave 5. Both these analyses (longitudinal and cross-sectional) will be conducted using a subsample of 5,210 core study members who took part in all waves with valid data related to loneliness and pet ownership. When we adjust our analysis for pet ownership in Wave 0 the sample size reduces to 2,141.

The answer to the Question 3 is investigated by creating loneliness pathways between Waves 2 to 5 and using this as an independent variable and pet ownership as the dependent variable. The role of socio-demographic risk factors for loneliness (Question 4) will be answered by developing multivariable models as part of our analysis of questions 1–3.

### Variables

#### Loneliness

In the English Longitudinal Study of Ageing loneliness is measured by the short form of the Revised University of California, Los Angeles (UCLA) loneliness scale in Waves 2 to 5. This instrument is a well-documented and widely used [55] and consists of three questions “How often do you feel you lack companionship”, “How often do you feel left out” and “How often do you feel isolated from others?” Responses are recorded on a 3-point Likert scale ranging from hardly ever/never, some of the time and often, resulting in a theoretical range of 3–9, with a higher score indicating greater loneliness. Score were dichotomised with those scoring 3–5 (three bottom quartiles) classified as “not lonely” and those with scores 6–9 (upper quartile) as “lonely” [11]. We used the short form UCLA loneliness scale in preference to a single-item loneliness measure (one question from CES-D questionnaire “Have you felt lonely much of the time during the past week?” with answers yes/no) because of concerns about the reliability of this measure with older people [21] as they may mask feelings of loneliness as consequence of its stigmatization [56] but also because the question is focused on loneliness in the last week which can be misleading and a potential source of under- or over-reporting.

#### Pet ownership

Pet ownership in both Wave 0 and 5 was measured using responses to the question “Do you keep any household pets inside your house/flat?” followed by questions asking whether they had a dog, cat, bird, other furry pet and other pet.

#### Covariates

Gender, age, marital status [57, 58], the presence of close personal relationships (social networks), social participation, working status, social position, household income, and health status were used as covariates in the analysis. Marital status was dichotomised into those never married/divorced/separated/widowed (not living with partner) and those living with partner (married/remarried/cohabiting). Information about social networks (family and friends) was available in all waves except Wave 0. A summary score was created to indicate whether the respondent had a close relationship with at least one of the following: spouse/partner, close family member or children and had at least one friend based upon responses to the following questions: about the number of family members and friends with whom respondent had contact, about the proximity of respondent’s marital relationship; and whether the positive support from the spouse, children, other relatives and friends was or was not present. Social participation was constructed as summary score from information about membership of any club, society, and church group or being an active member of neighbourhood community. Working status was derived from responses to questions about whether participants were (self-)employed, retired or did not work. For social status the short version of NS-SEC 3 category classification was used managerial/professional, intermediate and routine/manual. Household income was used categorised into quintiles [6].

Health related variables were also included in the analysis. A measure of immobility was constructed from difficulty in walking more than 200 yards (Wave 0) and difficulty walking more than ¼ of mile (other waves) [59]. Sensory impairment in Wave 0, derived variables about vision and hearing problems, were collected as binary measure with options “has condition” and “no condition”. In Waves 2 and 5 the answers to questions about impairments were on 5-point Likert scale, and dichotomised to be comparable with Wave 0. Self-rated health was classified using 5-point Likert score scale, dichotomised as good and poor health and used from the same waves as information about pet-ownership (Waves 0 and 5).

### Statistical methods

Frequency tables were constructed to describe the distribution of categorical variables in the individual waves of ELSA used in our analysis and mean age calculated for men and women for individual study waves.

The logistic regression analysis has three steps following our research questions. First, the role of pet ownership in Wave 0 (and in Wave 5 for cross-sectional analysis) as possible risk factor for loneliness in Waves 2 to 5 has been evaluated. A binary measure of loneliness was used as the dependent variable and pet ownership together with further covariates used as independent variables. Second, to consider pet ownership is a response to previous feelings of loneliness, we assessed if reported loneliness in Waves 2 to 5 affects pet ownership in Wave 5. Pet ownership was used as dependent variable and loneliness categorised as a binary variable the independent measure.

For both questions cross sectional analysis using data from Wave 5 was followed by prospective analysis in which the dependent variable was from later wave than independent variables. Thus for question 1 we looked at pet ownership in Wave 0 and loneliness at Wave 2, Wave 3, Wave 4 and Wave 5 (four separate prospective analyses). For question 2 three prospective and one cross-sectional analyses were conducted (loneliness in Wave 2, Wave 3, Wave 4, and Wave 5 and pet ownership in Wave 5). The number of prospective analyses was determined by the availability of data related to pet ownership and our measure of loneliness.

In step 3 the role of loneliness in future pet ownership was further assessed using pathways of loneliness. Pet ownership in Wave 5 was the dependent variable and loneliness pathway the independent variable. Our loneliness pathway was constructed as a combination of dichotomised UCLA loneliness variables in Waves 2, 3, 4 and 5, and a five-fold typology created: “always lonely”, “never lonely”, “pathway into loneliness”, “pathway out of loneliness” and “fluctuating pathway”.

In all three steps of our analysis, crude unadjusted odds ratio (OR) and 95% confidence interval (95% CI) were estimated, and this was followed by adjusted multivariable analysis to answer Question 4. Variables were tested as possible effect modifiers. Results are presented separately for men and women due to significant or borderline non-significant effect modification by sex. Missing data for the self-completed UCLA loneliness scale part of questionnaire ranged from 9.3% in Wave 2 and 11.5% in Wave 5 and was 1% for pet ownership questions. As the proportion of missing data was low we did not use imputation to increase analytical sample size [60].

Statistical analyses were carried out using STATA version MP 13.0

## Results

### Characteristics of the sample

Our analytical sample of 5,210 individuals was slightly younger in the first two waves than the main ELSA sample (61.4 years in Wave 0 compared with 63.4 for the main sample) but had higher mean age in later waves than the whole sample because the main dataset was boosted by new participants in consecutive waves. The gender distribution of the whole sample and analytical subsample is similar across all waves (43% of males and 57% of females) as is marital status (68.4% of our sample was married/living with partner while it was 66.9% in the whole ELSA sample). The proportion of widowed participants increased and proportion of married, remarried and those living with partner decreased by about 5% over 10 years of the study. The proportion of employed and not employed changed substantially and differs by nearly 30% between Wave 0 and Wave 5 which reflects the withdrawal of participants from the labour market.

A small percentage, 12%, of the sample had no children; 98% who had children reported a close relationship with them. Approximately one third of participants did not have spouse or partner with 97% of those who did reporting that they had a close relationship with them. Approximately 7.5% of participants did not have immediate family, and among those who had immediate family, approximately 13% did not have any close contact with them. Between 4 and 6% respondents did not have any friends and those who had them, 6% reported they do not have close relationship with them

A little over one-third, 39%, of ELSA participants owned pet while it was 41% in our sample. The rates of loneliness (as measured by the UCLA loneliness scale) increased very slightly from 18% to 20.6% over 10 years. Gender differences were stable over all the waves and rates of reported loneliness were about 7% higher in women compared with men (Table 1).

**Table 1:** **Descriptive characteristics of study sample**

	Wave 0 (2001)			Wave 2 (2004)			Wave 5 (2010)		
	All	Men	Women	All	Men	Women	All	Men	Women
**Total N**	**2,141**	946	1,195	**5,210**	2,272	2,938	**5,210**	2,272	2,938
**Mean age**	61.4	60.9	61.8	65.0	64.6	65.3	71.2	70.7	71.6
**Pet ownership** ^**3**^									
Yes (%)	41.1%	41.7%	40.7	NA	NA	NA	29.2%	29.8%	28.6%
**Ownership of house pet:**									
Dog (%)^1^	19.8%	20.3%	19.3%	NA	NA	NA	15.2%	15.6%	14.9%
Cat (%)^1^	21.7%	21.7%	21.7%	NA	NA	NA	13.3%	13.8%	12.9%
Bird (%)^1^	3.6%	3.7%	3.6%	NA	NA	NA	1.8%	1.7%	1.9%
Other furry pet (%)^1^	2.3%	2.3%	2.3%	NA	NA	NA	0.8%	1.0%	0.6%
Other pet (%)^1^	3.8%	4.8%	3.1%	NA	NA	NA	2.9%	3.4%	2.5%
**Loneliness** ^**3**^									
Yes (%)	NA	NA	NA	18.1%	14.1%	21.3%	20.6%	16.3%	23.9%
**Marital status**									
Single/divorced/separated/widowed (%)	31.6%	23.8%	37.9%	32.6%	22.4%	40.5%	36.7%	24.7%	46.0%
**Parenthood**									
Yes	NA	NA	NA	87.8%	87.2%	88.2%	87.9%	87.3%	88.4%
**Social participation** ^3^									
Yes	NA	NA	NA	22.6%	19.9%	24.5%	26.6%	25.1%	27.8%
**Close personal relationships** ^**3**^									
Yes	NA	NA	NA	99.8%	99.8%	99.9%	99.7%	99.6%	99.7%
**Working status** ^**3**^									
Working (%)	46.2%	52.9%	41.0%	33.7%	40.3%	28.6%	18.6%	24.1%	14.4%
**Social class**									
Managerial/professional	37.1%	45.0%	30.7%	34.3%	42.8%	27.6%	32.6%	40.4%	26.4%
Intermediate	24.3%	18.4%	29.1%	25.9%	20.0%	30.5%	27.1%	21.2%	31.8%
Routine/manual	38.6%	36.6%	40.3%	39.9%	37.3%	41.9%	40.3%	38.4%	41.8%
**Household income**									
1Q (low)	NA	NA	NA	16.5	10.9	20.8	18.6	14.4	21.8
2Q	NA	NA	NA	17.0	15.3	18.2	21.4	20.3	22.2
3Q	NA	NA	NA	19.4	19.8	19.1	21.3	21.1	21.5
4Q	NA	NA	NA	21.0	23.2	19.3	18.7	21.1	17.0
5Q (high)	NA	NA	NA	24.6	29.3	21.0	17.3	20.9	14.6
**Self-rated health** ^**2,3**^									
Poor	6.7%	7.5%	6.0%	23.0%	21.5%	24.1%	28.0%	26.7%	29.0%
**Long standing illness**									
Yes – limiting	32.2%	30.8%	33.4%	32.0%	28.8%	34.4%	38.6%	35.8%	40.7%
**Immobility** ^**3**^									
Some difficulty/much difficulty/unable to do test	11.0%	11.3%	10.8%	23.5%	19.7%	26.4%	35.7%	30.7%	39.6%
**Hearing difficulties** ^**3**^									
Yes	6.7%	8.5%	5.3%	4.2%	5.7%	3.1%	5.6%	7.1%	4.5%
**Seeing difficulties** ^**3**^									
Yes	3.4%	3.5%	3.3	2.8%	2.2%	3.3%	4.3%	3.0%	5.2%

^1^Some individuals owned more than one pet.

^2^In Wave 0 and in waves 1+ different categorization.

^3^ Binary variables; we show % of only one category.

### Question 1: pet ownership and later loneliness

#### Does pet ownership protect against loneliness?

In our cross-sectional analysis, those who reported pet-ownership in Wave 5 were 1.24 times more likely to report loneliness at the same time (see Table 2). In the prospective analysis those who reported pet-ownership in Wave 0 were 1.25 to 1.31 more likely to report loneliness in later waves. When the analysis was stratified by gender, as this is an effect modifier, having a pet increased reported loneliness 1.4-1.8 times in females (Table 2) after adjustment for all co-variates (age, marital status, working status, social class, health status, social inclusion, close personal relationships, and household income). This association was statistically significant for all analyses for women. The gender interaction was statistically significant, however, only in cross-sectional analysis when loneliness and pet-ownership were both measured Wave 5 and in one prospective analysis (Wave 0 to Wave 4). Although non-significant, the gender difference in the association between pet ownership and reported loneliness is very consistent (significant association in women and no association in men). We hypothesise that the non-significant interaction is a consequence of the smaller sample size in prospective analysis based upon reported pet ownership in Wave 0, where the number of participants is small compared to the other waves. Our analysis suggests that having a pet increased the likelihood of reporting loneliness among females in all adjusted analyses.

**Table 2:** **The association between pet ownership (in Wave 0 and Wave 5) and odds of loneliness (in Waves 2 to 5) (OR and 95% CI)**

	Loneliness				
	Cross-sectional	Prospective analysis			
Pet ownership	in wave 5	Wave 0-wave 2 (2001–2004)	Wave 0-wave 3 (2001–2006)	Wave 0-wave 4 (2001 – 2008)	Wave 0-wave 5 (2001–2010)
**N**	4,638	1,958	1,886	1,853	1,890
**All**					
** No**	1 (ref)	1 (ref)	1 (ref)	1 (ref)	1 (ref)
** Yes**	1.24 (1.06-1.47)	1.25 (0.98-1.61)	1.23 (0.96-1.58)	1.38 (1.08-1.77)	1.31 (1.03-1.68)
**Men**					
** No**	1 (ref)	1 (ref)	1 (ref)	1 (ref)	1 (ref)
** Yes**	1.04 (0.78-1.38)	1.03 (0.69-1.54)	0.98 (0.65-1.49)	0.84 (0.55-1.29)	1.06 (0.70-1.60)
**Women**					
** No**	1 (ref)	1 (ref)	1 (ref)	1 (ref)	1 (ref)
** Yes**	1.41 (1.15-1.73)	1.39 (1.01-1.92)	1.40 (1.03-1.90)	1.84 (1.34-2.52)	1.50 (1.09-2.05)
**P sex interaction**	*0.03*	*0.30*	*0.19*	*0.005*	*0.13*

Adjusted for gender (in pooled analysis), age, marital status, working status, social class, health status, social participation, close personal relationships, household income.

### Question 2: loneliness and later pet ownership

#### Is current pet ownership a response to previous feelings of loneliness?

In Wave 5 the odds of owning a pet were 25% higher for those who were lonely as compared to those who were not. This result is mainly accounted for by women who were lonely for whom the odds of owning a pet were almost 50% higher than their non-lonely counterparts (OR 1.3 to 1.8). Stratifying by pet ownership in Wave 0, the magnitude of the effect of loneliness on pet ownership in Wave 5 is larger among women who had pet at Wave 0 than among women who did not (for example, OR 2.02 and 1.52 for the relationship between loneliness at Wave 4 and pet ownership at Wave 5; not shown in the tables) but none of these interactions were significant. Therefore the relationship between loneliness and pet ownership in Wave 5 was adjusted for the pet ownership in Wave 0 (Table 3, “Adjusted 2”). Although the sex-specific effects are different (and of similar magnitude as “Adjusted 1”) the gender interactions are no longer statistically significant (except cross-sectional analysis) perhaps reflecting the reduced size of our analytical sample due to the limited availability of pet ownership data in Wave 0.

**Table 3:** **The association between reported loneliness (Waves 2 to 5) and odds of pet ownership (Wave 5) (OR and 95% CI)**

	Pet ownership			
	Cross-sectional	Prospective analysis		
Loneliness	Wave 5	Wave 2 to wave5 (2004–2010)	Wave 3 to wave 5 (2006–2010)	Wave 4 to wave 5 (2008–2010)
**N**	4,638	4,743	4,640	4,571
**Adjusted 1**				
**All**				
** No**	1 (ref)	1 (ref)	1 (ref)	1 (ref)
** Yes**	1.24 (1.05-1.46)	1.20 (1.01-1.42)	1.21 (1.02-1.43)	1.45 (1.23-1.72)
**Men**				
** No**	1 (ref)	1 (ref)	1 (ref)	1 (ref)
** Yes**	1.03 (0.77-1.36)	1.03 (0.76-1.38)	0.89 (0.67-1.20)	1.02 (0.77-1.36)
**Women**				
** No**	1 (ref)	1 (ref)	1 (ref)	1 (ref)
** Yes**	1.40 (1.14-1.72)	1.30 (1.05-1.61)	1.42 (1.16-1.74)	1.76 (1.43-2.17)
**P sex interaction**	*0.006*	*0.02*	*0.001*	*<0.001*
**Adjusted 2**				
**All**				
** No**	1 (ref)	1 (ref)	1 (ref)	1 (ref)
** Yes**	1.39 (1.01-1.91)	1.13 (0.8-1.59)	1.40 (1.01-1.96)	1.43 (1.02-2.01)
**Men**				
** No**	1 (ref)	1 (ref)	1 (ref)	1 (ref)
** Yes**	0.79 (0.47-1.34)	0.96 (0.58-1.59)	0.97 (0.57-1.65)	1.10 (0.65-1.87)
**Women**				
** No**	1 (ref)	1 (ref)	1 (ref)	1 (ref)
** Yes**	1.84 (1.26-2.68)	1.28 (0.86-1.89)	1.56 (1.07-2.27)	1.81 (1.22-2.67)
**P sex interaction**	*0.006*	*0.26*	*0.07*	*0.07*

Adjusted 1 = for gender(in pooled analysis), age, marital status, working status, social class, health status, social participation, close personal relationships, household income.

Adjusted 2 = additionally adjusted for pet ownership in Wave 0, N = 2,141.

### Question 3: loneliness pathways and later pet ownership

#### How do different pathways of loneliness influence current pet ownership?

Our final analysis evaluated how different pathways of loneliness affect pet ownership in Wave 5. The results are presented separately for men and women and are similar to previous analysis: no differences in the odds of pet ownership between different groups of men but significant between women who reported loneliness on all occasions or who moved out of loneliness and those who never reported loneliness. Those who always reported loneliness or moved out of loneliness were more likely to have pet in Wave 5 and these patterns were consistent when adjusted for pet ownership at Wave 0. Those who were persistently lonely were 2.4 times more likely to have a pet in Wave 5 than those who never reported loneliness whilst those who moved out of loneliness were 1.8 times more likely to have a pet than the non-lonely reference group (Table 4).

**Table 4:** **Pathways of loneliness and pet ownership in Wave 5 (OR and 95% CI)**

Loneliness pathway	Sample 1 (N)	Sample 2 (N) ^1^	Men		Women	
			Adjusted-sample 1	Adjusted-sample 2	Adjusted-sample 1	Adjusted-sample 2
**Never lonely**	2,540	1,025	1 (ref)	1 (ref)	1 (ref)	1 (ref)
**Always lonely**	256	84	0.81 (0.44-1.47)	1.12 (0.38-3.36)	1.41 (0.99-2.00)	2.40 (1.18-4.89)
**Into loneliness**	332	139	1.11 (0.76-1.62)	1.09 (0.59-2.04)	0.97 (0.70-1.35)	0.76 (0.41-1.40)
**Out of loneliness**	376	165	0.94 (0.60-1.48)	0.70 (0.31-1.60)	1.71 (1.25-2.35)	1.81 (1.02-3.20)
**Fluctuating**	333	137	1.02 (0.67-1.55)	1.00 (0.45-2.21)	1.24 (0.88-1.73)	1.28 (0.70-2.34)

Adjusted 1 = for gender (in pooled analysis), for gender (in pooled analysis), age, marital status, working status, social class, health status, social participation, close personal relationships, household income.

Adjusted 2 = additionally adjusted for pet ownership in Wave 0 = Sample 2.

^1^Those, who have information on pet ownership in Wave 0.

## Discussion

Levels of reported loneliness in ELSA are approximately 19% and these are roughly stable over time. Women reported loneliness more frequently than men (a differential of around 7%), and this difference is also approximately stable over time and supports previous studies using the revised UCLA scale [61] but no other studies using other scales to measure loneliness which reports higher rates of loneliness for men [62]. Loneliness in ELSA is reported more frequently in comparison with European data from the SHARE study conducted across a range of European countries (more information on http://www.share-project.org/). The reported rates of loneliness in SHARE in European countries (measured by the same instrument as in ELSA study and in comparable years) are somewhat lower oscillating between 5% (in Denmark or Switzerland), 13% in France and 18% in Hungary [2] compared with 20% for ELSA. These differences in the prevalence of loneliness across Europe support the hypothesis that loneliness is culturally defined and is associated with expectations. For example the prevalence of loneliness in Greece is traditionally reported as one of the highest in the Europe but only about 5% of older people live alone and about 60% reported that they had close daily contacts with family members or friends [1, 63].

The goal of our study was to assess the relationship between loneliness and having a pet. In particular we wanted to explore the direction of the association and answer the question whether pet ownership is a protection against or response to loneliness? Having a pet was reported by 41% of respondents at baseline (Wave 0) and by nearly 30% in Wave 5 and more than one pet by 24% and 18% of respondents respectively. We do not know why pet ownership decreased but it seems plausible that this may reflect reduced income resulting from retirement; deteriorating health resulting in pet care being too demanding and the death of pets.

We demonstrate that those who reported that they had a pet at the beginning of the study were 1.2-1.4 more likely to report loneliness compared to those who did not. This overall association masks a significant gender effect being confined to women but not men. Looking at the relationship the other way around and focusing upon loneliness as a predictor of pet ownership we see a similar association of a similar magnitude. In pooled analysis those who reported loneliness were 1.2-1.5 times more likely to have a pet at follow up. Again the gender interaction was significant in all analyses demonstrating that the association between loneliness and pet ownership is confined to women. Our pathway analysis demonstrated that women who always reported being lonely and those who moved out of loneliness were more likely to report pet ownership than their non-lonely contemporaries (odds ratios of 2.4 and 1.8 respectively). These results mean, perhaps, that pet ownership can be a response to loneliness for the always lonely and a protection for those who recovered from loneliness.

Gender is the key factor in our analysis. Our results suggest that the association between pet ownership and loneliness is particularly strong in women. Age, social, demographic and health behaviours variables including established risk factors for loneliness (such as age or marital status) do not substantially affect the magnitude or direction of the association between loneliness and pet ownership.

There are, of course, some limitations to our study. Loss to follow-up of individuals between the waves of ELSA data collection might have introduced selection bias. Recent articles using ELSA data suggest that sample attrition is, for example, greater among those who were in a disadvantaged socioeconomic position at the start of the study but any bias due to attrition might be only small [64]. There was a relatively small subsample of participants, who were asked about pet ownership in Wave 0 of ELSA. We do not have information about how long participants had owned a pet or whether they looked after somebody else’s pet. We also do not have sufficient consecutive information to determine the relation between the initial reporting of loneliness, possible acquisition of a pet and the subsequent loneliness status to see the whole sequence of events to explore reverse causality in full detail. We could not adjust for seasonality and although loneliness is higher in spring and winter [65, 66] we do not think that this would significantly alter our results. Finally, as we performed relatively large number of hypothesis tests, we focused more on the magnitude of the effects when interpreting the results rather than just purely focusing on significance of findings.

## Conclusions

It is commonly assumed that pet ownership ‘protects’ older people against loneliness. Our analysis has demonstrated that, for women, this may be a plausible hypothesis as it is associated with recovery from loneliness. However we have also demonstrated that for women who are always lonely pet ownership may be a response to their loneliness. Our results contribute to research on loneliness by demonstrating the complexity of the link between pet ownership and loneliness-it can be both a response to loneliness and a potential pathway out of loneliness. We also demonstrate that these relationships are only demonstrated by women and are not moderated by established loneliness risk factors or confounders.

These results suggest a number of areas for future research and have implications for policy and practice. There is considerable scope for qualitative research examining the issue of pet ownership in later life in more detail and how older people see this as a response or pathway out of loneliness. There is a clear need for such research to explore the important gender dimension identified in our analysis. Quantitative studies can demonstrate a link between gender, loneliness and pet ownership but we need to conduct qualitative research to explore the factors that account for these relationships. In addition these results caution us as to the appropriateness of pet based therapies and interventions against loneliness. We may speculate that, based on our findings, that such interventions may be more appropriate and acceptable to women than men. Again we need further research to explore the nature of the relationships between gender, loneliness and pet ownership in order to develop interventions that are appropriate, acceptable and effective.

## Abbreviations

ELSA: English Longitudinal Study of Ageing
AAT: Animal assisted therapy
UCLA: University of California, Los Angeles
SHARE: The Survey of Health, Ageing and Retirement in Europe
OR: Odds ratio
CI: Confidence interval.
